# Instructional educational games in pharmacy experiential education: a quasi-experimental assessment of learning outcomes, students’ engagement and motivation

**DOI:** 10.1186/s12909-023-04742-y

**Published:** 2023-10-11

**Authors:** Mariam Dabbous, Fouad Sakr, Jihan Safwan, Marwan Akel, Diana Malaeb, Mohamad Rahal, Anwar Kawtharani

**Affiliations:** 1 School of Pharmacy, Lebanese International University, Beirut, Lebanon; 2 School of Education, Lebanese International University, Beirut, Lebanon; 3 UMR U955 INSERM, Institut Mondor de Recherche Biomédicale, Université Paris-Est Créteil, Créteil, France; 4 École Doctorale Sciences de la Vie et de la Santé, Université Paris-Est Créteil, Créteil, France; 5 INSPECT-LB: Institut National de Santé Publique, Épidémiologie Clinique et Toxicologie-Liban, Beirut, Lebanon; 6 International Pharmaceutical Federation (FIP), The Hague, The Netherlands; 7 College of Pharmacy, Gulf Medical University, Ajman, UAE

**Keywords:** Educational games, Instructional games, Experiential education, Learning outcomes, Competencies, Motivation, Engagement

## Abstract

**Background:**

This study aimed to determine the impact of implementing instructional educational games on attaining the intended learning outcomes mapped with the competencies of a pharmacy practice experience course, and to assess students’ attitudes towards motivation and engagement in this active learning activity.

**Methods:**

This was a quasi-experimental study that utilized a pretest-posttest for the research groups. Students were divided into teams and challenged to answer different questions related to the case scenarios. Different gaming platforms as Gamilab, Wisc-Online, and Quizizz were accordingly used to create different questions that help students memorize medications’ brand names, and acquire the advanced community knowledge and skills. The attainment of the intended learning outcomes was assessed and compared between the experimental and control groups through the course total average of grades, and the subsequent averages of domains relating to the course competencies. Attitudes towards motivation and engagement in educational games activities were also assessed among the experimental group.

**Results:**

A total of 233 students were enrolled in the study. The experimental group had significantly higher total posttest average compared to the control group (Beta = 7.695, 95% CI = 4.964–10.425, P < 0.001). The experimental group had also significantly higher averages of competency domains related to foundational knowledge (Beta = 1.471, 95% CI = 0.723–2.219, P < 0.001), pharmaceutical care (Beta = 1.650, 95% CI = 0.673–2.627, P < 0.001), essentials to practice and care (Beta = 1.838, 95% CI = 0.626–3.050, P < 0.003), and approach to practice and care (Beta = 2.736, 95% CI = 1.384–4.088, P < 0.001) averages. The experimental group reflected positive attitudes toward gamification engagement and motivation, with greater than 60% of the students recommend engage educational games to be part of the course.

**Conclusion:**

Incorporation of educational games into pharmacy practice experiences resulted in better learning outcomes. This kind of active learning appears to be acceptable and motivational for students, and is recommended for further research in didactic courses in the pharmacy curriculum.

## Background

Incorporating innovative active learning strategies to encourage students’ engagement, motivation, and knowledge retention have been challenging in pharmacy education [1]. In fact, the core element of pharmacy curriculum is traditional didactic teaching that is considered static and boring for the Z generation, who are born in the digital age of online games [2]. The limited use of innovative teaching techniques in pharmacy education with widespread use of teacher-centered strategies led to a lower student engagement and interaction, decreased motivation, lower level of understanding, and increased absence rate in classroom [3]. This raises the need of refinement of teaching methods, and implementation of new pedagogical methods in pharmacy curriculum to enhance learner motivation, engagement, and knowledge retention.

The school of pharmacy (SOP) at the Lebanese International University (LIU) has developed a contemporary educational philosophy curriculum with a set of competencies and intended learning outcomes that enable students to proactively function as learners in dynamic and complex professional pharmacy practice settings [4]. This cannot be simply achieved by traditional didactic methods, but rather by adopting more effective methods of teaching and learning. Therefore, the SOP employs different teaching and learning methods to achieve the program learning outcomes and develop students’ competencies [5].

Educational games have evolved over the past 20 years. The journey started with CD-ROM games to help students to prepare for class time, and progressed to reach today virtual and augmented reality that help educators to engage students innovatively in scholastic activities [6]. Indeed, integrating technology in the classroom expands the ability to create learning opportunities that match the students’ multiple learning styles [7]. Instructional educational games are one of the interactive tools that amassed increased attention in academia as it presents an important solution to respond to the raised needs. They are one of the active learning strategies that help students explore, create, imagine, interact, role play, and learn on a more effective, entertaining and vivid platform [8]. They also allow the learner to engage in a competitive activity with predetermined rules to enhance students’ communication, collaboration, critical-thinking skills, and knowledge acquisition and retention [9]; thus supporting higher-level of thinking and discussions which are crucial to the pharmacist [10, 11]. Educational games also enable educators to create a realistic setting with real life scenarios promoting students to practice safely [12].

Game-based learning has emerged as a promising approach to enhance experiential education in pharmacy. It offers an interactive and engaging way of learning, promoting students’ motivation, knowledge retention, and skills development [13]. Despite the use of educational games within the education context and in different disciplines as science, technology, engineering, math, and health professions [14], there have been few studies of gamification in pharmacy, and published research in this area is still sparse [15]. Introducing gamification in pharmacy curriculum aims to improve students’ knowledge retention, motivation, and engagement. Research on game-based learning in pharmacy education is necessary to explore its effectiveness, best practices, and potential impact on students’ learning outcomes. Therefore, conducting research on game-based learning in experiential pharmacy education is essential to inform educational practices and optimize learning outcomes. This quasi-experimental study aimed to determine the impact of implementing instructional educational games in a pharmacy practice experience (PPE) course on acquiring the intended learning outcomes mapped with the desired competencies of the course. It also aimed to assess students’ attitudes around motivation and engagement in this active learning activity.

## Methods

### Study design and intervention

This was a quasi-experimental study that utilized a pretest-posttest for the research groups. Recruitment was carried out by sending an invitation via email to all students who are registered in the course to participate in the study. The first group or the experimental group included students who voluntarily engaged in instructional gamifications during the course, whereas the second group or the control group included the students who opted not to participate. Accordingly, two homogenous groups of experimental and control teams were established.

### Learning objectives

LIU offers a set of PPE courses during the first, second and third professional years [16]. We previously described the LIU model of experiential education in pharmacy curriculum [17]. This study took place in the PPE of the second professional year, which includes advanced topics related to patient care in community settings. The course is divided into 3 modules, and each module is divided into different topics related to non-communicable and communicable diseases. The course learning outcomes are intended to provide adequate knowledge and skills to engage students in patient care for diverse population, and interact with healthcare professionals. The course equips the students with competencies related to 4 domains. Domain 1 (D1) “Foundational Knowledge” allows students to apply knowledge acquired through didactic and simulated courses to make a therapeutic decision in a real practical setting. Domain 2 (D2) “Pharmaceutical Care” enables students to assess medication use based on evidence-based medicine and rely on patient profile, as well as to compound and choose the correct dosage form and dispense medications while counseling patients. Domain 3 (D3) “Essentials to Practice and Care” permits students to utilize all available resources in order to provide and optimize patient-centered care, as well helps students to know how to promote patient and population health. Domain 4 (D4) “Approach to Practice and Care” empowers students to develop the knowledge, skills, abilities, behaviors, and attitudes necessary to solve problems, educate, advocate, collaborate, and conduct research while working with people from diverse backgrounds, as well as effectively communicate verbally and nonverbally [18].

### Course delivery

During the ongoing COVID-19 pandemic, the practice experience courses involved hybrid learning in which students followed a structured course manual to prepare various practice topics through valid information retrieval. The course engaged students in patient educational activities, PowerPoint presentations on community topics, and discussions on different cases with preceptors. All activities were done virtually while students practiced for several hours in a community pharmacy.

### Educational strategies and materials

Students played the role of the pharmacist through virtual meetings that took place via Google Meet with the course coordinator every other week over a 12-week course period. Separate meetings throughout the course were organized for each of the experimental and control groups. Gamified learning activities involved exclusively the experimental group, whilst the control group continued with traditional learning activities. Students were divided into teams and challenged to answer different questions related to the case scenarios, and the team that answered first earned points, and each time the winner was the team with higher points. Different gaming platforms as Gamilab, Wisc-Online, and Quizizz were accordingly used to create different questions that help students memorize medications’ brand names, and acquire the advanced community knowledge and skills in a fun and competitive way. Students accessed all these gaming platforms on their pace weekly and got immediate feedback. The case scenarios and questions were related to the course intended learning outcomes that are mapped with the previously described four domains.

### Procedures

The baseline characteristics of the research groups were retrieved from the university management system for all students who are registered in the course. The averages of the grades at the beginning of the course before gamification “pretest” and by the end of the course after gamification “posttest” were used to assess and compare attainment of the intended learning outcomes between the experimental and control groups. The pre- post-testing involved a standardized examination for both groups, who continued the common teaching methods of the course irrespective of their group allocation to avoid the risk of information bias from the control group. The time between the pre- and post-test assessment was 12 weeks. Moreover, the experimental group responded to a questionnaire that aimed to determine the level of motivation and engagement among participants. The questionnaire included two parts. The first part tackled the sample description and the sociodemographic data of students relating to age, gender, Grade Point Average (GPA), and area of residence. The second part included 14 items that were adapted from the active learning motivation assessment scale, which is a validated and reliable scale to assess motivation to engage in game-based learning and possibly other active learning activities [19]. All items involved a five-point Likert scale ranging from “strongly disagree” to “strongly agree” around the general characteristics of instructional game-playing, engagement and motivation, and the ability of this learning tool to fulfill its anticipated outcomes.

### Ethical aspects

All methods were performed in accordance with the relevant guidelines and regulations. The study protocol was approved by the Research and Ethics Committee of the School of Pharmacy at the Lebanese International University (protocol number: 2020RC-055-LIUSOP), who waived the need for a written informed consent.

### Statistical analysis

Data were collected via Google Forms, then extracted into Microsoft Excel and analyzed by IBM Statistical Package for the Social Sciences (SPSS version 26). Descriptive statistics were evaluated by means and standard deviation (SD) for continuous variables including age, GPA, total average, and averages of D1, D2, D3, and D4. Gender was a categorical variable (1 = male, 2 = female), and it was evaluated by its frequencies and percentages. The comparison of the baseline characteristics of the research groups utilized independent sample T-test for the continuous variables and chi-square analysis for the categorical variables. The pre- post-test course averages were compared between each of the experimental and control groups through paired sample T-test. The normal distribution of class averages was confirmed by histogram and Shapiro-Wilk test. Afterwards, five models of multiple linear regression were conducted taking the study group (experimental versus control) as independent variable, while adjusting for age, gender, and GPA as potential confounding factors. The first model included the final (post-test) course total average as the dependent variable. The remaining 4 models including the final (post-test) averages of D1, D2, D3, and D4 as dependent variables respectively. Results were reported as unstandardized Beta with 95% confidence interval (CI), and the level of significance was set at P < 0.05 with an acceptable margin of error = 5%.

## Results

### Sample and baseline characteristics

A total sample of 233 students were enrolled including 69 students (29.6%) in the experimental group and 164 students (70.4%) in the control group producing a ratio of 1:2.4. The characteristics of the sample were comparable at baseline for the experimental and control groups, except for the average of D3 that was higher in the experimental group. The mean age of the total sample was 23.16 (± 1.82), 70% were females, and the mean GPA was 3.14 (± 0.35). The pretest total course average was 65.59 (± 18.86), and the averages of the subsequent domains were 13.68 (± 4.15) for D1, 13.79 (± 4.15) for D2, 17.53 (± 7.07) for D3, and 20.59 (± 6.62) for D4. The full characteristics of the sample and research groups at baseline are shown in Table 1.

**Table 1:** Sample and baseline characteristics

Variable	Total sample N = 233 Mean (SD) or N (%)	Experimental group N = 69 (29.6%) Mean (SD) or N (%)	Control group N = 164 (70.4%) Mean (SD) or N (%)	P value
**Age**	23.16 (1.82)	23.49 (2.05)	23.02 (1.7)	0.075
**Gender**				0.243
• Male • Female	70 (30.0) 163 (70.0)	17 (24.6) 52 (75.4)	53 (32.3) 111 (67.7)	
**GPA**	3.14 (0.35)	3.12 (0.40)	3.14 (0.34)	0.707
**Pre-gamification course average**				
Total average • Domain 1 average ^a^ • Domain 2 average ^b^ • Domain 3 average ^c^ • Domain 4 average ^d^	65.59 (18.86) 13.68 (4.15) 13.79 (4.15) 17.53 (7.07) 20.59 (6.62)	66.86 (20.10) 13.27 (4.51) 13.73 (4.27) 19.23 (7.18) 20.64 (6.93)	65.04 (18.34) 13.85 (4.0) 13.82 (4.11) 16.79 (6.91) 20.58 (6.50)	0.513 0.347 0.875 0.019 0.950

^a^ Domain 1: “Foundational Knowledge”

^b^ Domain 2: “Pharmaceutical Care”

^c^ Domain 3: “Essentials to Practice and Care”

^d^ Domain 4: “Approach to Practice and Care”

### Comparison of pre- post-test course averages of each of the experimental and control groups

The experimental group had significantly higher posttest total average with a mean difference of 14.79 (± 22.43) compared to the pretest average (P < 0.001). The posttest averages of competency domains were also significantly higher than the pretest averages for D1 (mean difference = 4.17 (± 5.15), P < 0.001), D2 (mean difference = 1.52 (± 4.81), P < 0.013), D3 (mean difference = 4.62 (± 8.51), P < 0.001), and D4 (mean difference = 4.49 (± 8.46), P < 0.001).

The control group had also significantly higher posttest total average with a mean difference of 9.49 (± 19.13) compared to the pretest average (P < 0.001). The posttest averages of competency domains were also significantly higher than the pretest averages for D1 (mean difference = 2.14 (± 4.46), P < 0.001), D3 (mean difference = 5.20 (± 7.56), P < 0.001), and D4 (mean difference = 2.25 (± 7.10), P < 0.001). No significant difference was found between the pre- and post-test averages of D2 in the control group (mean difference = 0.10 (± 5.11), P = 0.813). The comparison of the pre- post-test course averages of the experimental and control groups is shown in Table 2.

**Table 2:** Comparison of the pre- post-test course averages of the experimental and control groups

Variable	Pre-test Mean (SD)	Post-test Mean (SD)	Mean difference (SD)	P value
**Experimental group**				
Total average	66.86 (20.1)	81.65 (9.74)	14.79 (22.43)	< 0.001
Domain 1 average ^a^	13.27 (4.52)	17.44 (2.41)	4.17 (5.15)	< 0.001
Domain 2 average ^b^	13.73 (4.27)	15.24 (3.19)	1.52 (4.81)	0.013
Domain 3 average ^c^	19.23 (7.18)	23.85 (4.37)	4.62 (8.51)	< 0.001
Domain 4 average ^d^	20.64 (6.93)	25.12 (4.44)	4.49 (8.46)	< 0.001
**Control group**				
Total average	65.04 (18.34)	74.53 (9.74)	9.49 (19.13)	< 0.001
Domain 1 average ^a^	13.85 (3.99)	15.99 (2.59)	2.14 (4.46)	< 0.001
Domain 2 average ^b^	13.82 (4.11)	13.73 (3.30)	0.10 (5.11)	0.813
Domain 3 average ^c^	16.79 (6.91)	21.99 (4.25)	5.20 (7.56)	< 0.001
Domain 4 average ^d^	20.58 (6.50)	22.82 (4.66)	2.25 (7.10)	0.001

^a^ Domain 1: “Foundational Knowledge”

^b^ Domain 2: “Pharmaceutical Care”

^c^ Domain 3: “Essentials to Practice and Care”

^d^ Domain 4: “Approach to Practice and Care”

### Impact of educational games on class averages

Five models of multivariable linear regression were conducted taking the post-test course total competency domains averages as the dependent variable. The experimental group had significantly higher total posttest average compared to the control group (Beta = 7.695, 95% CI = 4.964–10.425, P < 0.001). Students with higher GPA had also higher total posttest average compared to students with lower GPA (Beta = 10.949, 95% CI = 7.102–14.796, P < 0.001). There was no significant association between age and gender with a higher or lower total posttest average. The posttest averages of all of the 4 competency domains were also significantly higher among participants in educational games. The experimental group had significantly higher averages of D1 (Beta = 1.471, 95% CI = 0.723–2.219, P < 0.001), D2 (Beta = 1.650, 95% CI = 0.673–2.627, P < 0.001), D3 (Beta = 1.838, 95% CI = 0.626–3.050, P < 0.003), and D4 (Beta = 2.736, 95% CI = 1.384–4.088, P < 0.001). Table 3 presents five models of multiple linear regression taking the course total average and the averages of competency domains as dependent variables.

**Table 3:** Multiple linear regression taking the course total average and the averages of competency domains as dependent variables

Independent variable	Beta	95% confidence interval	P value
**Model 1: taking the total posttest average as dependent variable**			
Group (Experimental vs. Control)	7.695	4.964; 10.425	< 0.001
Age	-0.100	-0.872; 0.671	0.798
Gender (Male vs. Female)	2.818	0.005; 5.630	0.050
GPA	10.949	7.102; 14.796	< 0.001
**Model 2: taking the posttest average of D1 (Foundational Knowledge) as dependent variable**			
Group (Experimental vs. Control)	1.471	0.723; 2.219	< 0.001
Age	-0.185	-0.397; 0.026	0.085
Gender (Male vs. Female)	-0.049	-0.820; 0.721	0.900
GPA	1.214	0.160; 2.267	0.024
**Model 3: taking the posttest average of D2 (Pharmaceutical Care) as dependent variable**			
Group (Experimental vs. Control)	1.650	0.673; 2.627	0.001
Age	-0.110	-0.386; 0.166	0.432
Gender (Male vs. Female)	0.642	-0.365; 1.648	0.210
GPA	1.754	0.377; 3.131	0.013
**Model 4: taking the posttest average of D3 (Essentials to Practice and Care) as dependent variable**			
Group (Experimental vs. Control)	1.838	0.626; 3.050	0.003
Age	0.131	-0.211; 0.474	0.451
Gender (Male vs. Female)	0.670	-0.578; 1.919	0.291
GPA	4.628	2.920; 6.336	< 0.001
**Model 5: taking the posttest average of D4 (Approach to Practice and Care) as dependent variable**			
Group (Experimental vs. Control)	2.736	1.384; 4.088	< 0.001
Age	0.064	-0.318; 0.446	0.741
Gender (Male vs. Female)	1.555	0.162; 2.948	0.290
GPA	3.354	1.449; 5.259	0.001

### Attitudes of students on gamification in experiential learning

The majority of students in the experimental group reflected positive attitudes towards gamification in experiential education. More than 67% agree to strongly agree that this educational gamification is beneficial to acquire the intended learning outcomes of the course and the desired knowledge easier. More than 58% agree to strongly agree that the games helped them to develop confidence in the subject area and skills that apply to their academic career and/or professional life. Furthermore, 60% reported that the games motivated them to learn the course material more than class activities that did not use the game; and more than 60% recommend educational games to be part of the course next year. The detailed attitudes of students are reported in Table 4.

**Table 4:** Attitudes of students on gamification in experiential education

Variable^*^	Strongly Agree (%)	Agree (%)	Neutral (%)	Disagree (%)	Strongly Disagree (%)
Beneficial to acquire the desired knowledge easier.	31.6	41.1	23.2	4.2	0
Beneficial to acquire the intended learning outcomes of the course.	26.3	41.1	30.5	2.1	0
Beneficial to incorporate additional information that was not to be acquired without the games.	24.2	40	32.6	3.2	0
Enhanced the online learning experience and made it easier.	27.4	32.5	37.9	2.1	0
Helped me to apply the course content to solve clinical problems.	23.2	41.1	32.6	3.2	0
Helped me learn the course content.	22.9	42.7	30.2	4.2	0
Helped me connect ideas in new ways.	26.3	43.2	27.4	3.2	0
Helped me to participate in the course activity in ways that enhanced my learning.	26.3	45.3	27.4	1.1	0
Helped me to develop confidence in the subject area.	15.8	48.4	33.7	2.1	0
Helped me to develop skills that apply to my academic career and/or professional life.	17.9	40	38.9	3.2	0
Motivated me to learn the course material more than class activities that did not use games.	25.3	34.7	33.7	5.3	1.1
Provided me with the opportunity to practice and improve my competencies.	23.2	47.4	26.3	3.2	0
My attention to the task(s) was greater than with traditional modules.	16.8	31.6	40	11.6	0
Important supplement to this class.	24.2	41.1	32.6	2.1	0
I recommend the pharmacy games to be part of the course next year.	31.6	29.5	35.8	3.2	0

^*^Total number of participating students = 69

## Discussion

This study investigated the impact of instructional educational games on the learning outcomes of experiential education through a quasi-experimental design involving pre- post-testing, and assessed students’ attitudes toward this learning activity. The outcomes were evaluated through a standard common examination for the experimental and control groups, and comparability of the academic performance of the study groups was verified at the baseline. Better course outcomes were determined with the incorporation of educational games, with positive attitudes reflected by the participating students.

### Learning outcomes

The participants in the experimental had significantly higher post-testing average compared to non-participants. Instructional games therefore appear to have a significant impact to acquire the desired learning outcomes and competencies in pharmacy practice experiences. Our results are consistent with the findings of other studies that reported higher quiz scores in the group who utilized gaming as an added tool in an introductory pharmacy practice experience (IPPE) course [20]. However, that study involved only the low order of thinking as it included items and gaming materials that necessitate rote memorization. The present study incorporated items that involve both knowledge and memorization, targeting low order of thinking, and case scenarios challenged questions that tackle higher order of thinking. The higher order of thinking involved dealing with real patient case situations in order to analyze patient data, evaluate and select drug therapy, and create a patient care plan. Educational games evidently promote active learning, which afterward improve students’ overall knowledge and performance [21]. Our findings support the literature by determining a significant role of educational games in obtaining knowledge and acquiring skills in experiential education of pharmacy.

The research groups were evaluated through standard common examinations that were categorized into 4 domains as adapted by the competency-based curriculum of the SOP at the LIU. We determined the impact of gamification on each of the four domains categorized into foundational knowledge, pharmaceutical care, essentials to practice and care, and approach to practice and care. The experimental group had significantly higher posttest averages for all domains compared to the control group. The gamification activities helped students in gaining foundational knowledge and facilitated their memorization to medications’ brand names. Moreover, students applied pharmaceutical mathematics to perform accurate medication calculations. The case scenarios allowed students to practice on providing effective health and medication information to patients or caregivers focusing on important counseling tips, adverse events, and drug interactions. Students collaborated with peers and worked as a team to provide the effective counseling services. Although no studies assessed the impact of educational games on competency domains, our results are harmonious with other findings, which determined that educational games helped students to develop long term memories, improve knowledge, promote teamwork and enhance communication skills [8, 22, 23]. Educational games reportedly enhance participants’ performance through different cognitive dimensions focusing on memory, understanding and conceptual application [24]. The current study adds to the literature that educational games allow students to acquire all of the intended learning outcomes and desired competencies in an experiential pharmacy education course.

This study also assessed the impact of the course itself on the intended learning outcomes. Our results showed that the course had a significant impact on knowledge and skill acquisition, as determined by the pre- post-test comparison of the averages for the control group. Nonetheless, our multiple linear regression determined that incorporation of gamification into the PPE course is associated with significantly better learning outcomes. This was reflected by higher posttest averages, which was statistically significant for the total and all 4-domain averages.

Our findings are consistent with the normal trend of academic performance where students with higher GPA achieve better outcomes [25]. However, this doesn’t interfere with the results of the present study as the difference in the mean pretest GPA between the experimental and control groups was minimal and statistically not significant. Moreover, our results do not appear to be confounded by the sociodemographic characteristics of the students, as the multiple linear regression analysis has shown no significant association between age, gender, or area of residence and learning outcomes. Though residual confounders relating to digital literacy, stress level, and time management cannot be completely precluded as they weren’t assessed because they are beyond the scope of this research.

### Attitudes toward motivation and engagement

Our students demonstrated positive attitudes towards participation in this educational activity. The consistent weekly, voluntarily, and ungraded participation reflects a high level of engagement and motivation to participate in educational gaming activities. Instructional educational games appear to have a positive impact on the students’ perception around acquiring the desired knowledge and skills. Students feel motivated to participate in this educational activity as it develops confidence in the subject area, and motivates learning more than routine class activities. Our results are consistent with other findings that reported students’ engagement and satisfaction with educational games, in addition to confidence in knowledge and abilities [26–28]. In the age of technology integration, educational games boost the motivation and interaction of learners throughout their educational journey, and creates a fun and interactional learning environment [29]. Game mechanics including points, leader boards, and immediate feedback are important features in educational games, and play an essential role in fostering learners’ motivation and engagement [30–32]. These elements were integrated in each gaming activity of this research to instill a sense of competition among students through earning points and celebrating the winning team. Similarly, a leader board was shared after each module for all students to see their names progress as a result of their accomplishments. Students were able to receive immediate feedback, and this was a principal factor in the learners’ knowledge and engagement during the activities.

### Strengths and limitations

This study has several strengths. The quasi-experimental design confirms the impact of gamification on the learning outcomes, and provides internal validity to our derived conclusions. The results are reliable as the measurement tool was a valid standard common examination that is routinely utilized in the course assessment. This common objective assessment also minimized the risk of any possible selection or information bias. Volunteering into the experimental group also minimized the risk of information bias because it precluded possible preset negative attitudes that may result from compulsory participation since instructional educational games were not yet a standard part of the course syllabus. On the other hand, our findings have a low external validity as the study was conducted in a PPE course during the second professional year of the BPharm program only. The routine examination may also not be the perfect assessment tool for all domains to determine their outcomes. Further research in this context is suggested to determine the impact of instructional educational games on the outcomes of didactic courses, and thus determine generalizability of the results. Moreover, voluntary participation and student self-selection into the study groups may have been associated with a possible risk of selection bias. However, this bias is minimized as the two groups were academically comparable and there was no statistically significant difference in the baseline characteristics of the students. Finally, the assessment of attitudes toward motivation and engagement in educational games utilized primarily descriptive analysis. Further research is still recommended to analyze the correlation between these factors and their impact on the learning outcomes.

### Implications for practice

Pharmacy graduates are expected to have adequate knowledge and experiences on frequently encountered community conditions [33–39]. They are also expected to demonstrate professional skills to promote the public health [40–44]. The necessitates a comprehensive pharmacy curriculum with innovative practice experiences [45–47]. The current study determined a significant positive role of instructional educational games on pharmacy practice experiences. The findings add to the literature that educational games allow students to acquire the intended learning outcomes mapped with the desired competencies relating to foundational knowledge, pharmaceutical care, essentials to practice and care, and approach to practice and care. This research reveals that instructional games in experiential education can provide preceptors and faculty members with an added pedagogical method for experiential learning, and render concepts simpler to teach and comprehend. In this context, several questions emerge regarding the future research directions. For instance, the extent to which game-based learning can be relied upon as an alternative to traditional learning in experiential pharmacy education is an area of inquiry. Additionally, exploring the applicability of this active learning style in didactic pharmacy education is another aspect to be considered. Further research is highly recommended to gain deeper insights and provide more comprehensive answers to these inquiries.

## Conclusion

Up to the best of our knowledge, this is the first study to determine the impact of instructional educational games on the learning outcomes of pharmacy experiential education in Lebanon. Incorporation of gamification into pharmacy practice experiences resulted in better learning outcomes, as reflected by higher acquisition of foundational knowledge and skills for pharmaceutical care, essentials to practice and care, and approach to practice and care. Gamification provide an additional educational tool for preceptors and faculty members to enhance the student learning experience. They also render concepts simpler to teach and comprehend, resulting in increased student concentration and knowledge acquisition. This kind of active learning appears to be acceptable and motivational for students, and is recommended for further research in didactic courses in the pharmacy curriculum.

## Data Availability

The datasets used and/or analysed during the current study are available from the corresponding author on reasonable request.

## List of abbreviations

SOP: school of pharmacy
LIU: Lebanese International University
PPE: pharmacy practice experience
D1: domain 1
D2: domain 2
D3: domain 3
D4: domain 4
GPA: Grade Point Average
SPSS: Statistical Package for the Social Sciences
CI: confidence interval
IPPE: introductory pharmacy practice experience
